# All oxide based flexible multi-folded invisible synapse as vision photo-receptor

**DOI:** 10.1038/s41598-023-28505-3

**Published:** 2023-01-26

**Authors:** Ping-Xing Chen, Debashis Panda, Tseung-Yuen Tseng

**Affiliations:** 1 Institute of Electronics, National Yang-Ming Chiao Tung University, Hsinchu, 30010 Taiwan; 2 Department of Electronics and Communication Engineering, CV Raman Global University, Bhubaneswar, 752054 India

**Keywords:** Engineering, Nanoscience and technology

## Abstract

All oxide-based transparent flexible memristor is prioritized for the potential application in artificially simulated biological optoelectronic synaptic devices. SnO_x_ memristor with HfO_x_ layer is found to enable a significant effect on synaptic properties. The memristor exhibits good reliability with long retention, 10^4^ s, and high endurance, 10^4^ cycles. The optimized 6 nm thick HfO_x_ layer in SnO_x_-based memristor possesses the excellent synaptic properties of stable 350 epochs training, multi-level conductance (MLC) behaviour, and the nonlinearity of 1.53 and 1.46 for long-term potentiation and depression, respectively, and faster image recognition accuracy of 100% after 23 iterations. The maximum weight changes of -73.12 and 79.91% for the potentiation and depression of the synaptic device, respectively, are observed from the spike-timing-dependent plasticity (STDP) characteristics making it suitable for biological applications. The flexibility of the device on the PEN substrate is confirmed by the acceptable change of nonlinearities up to 4 mm bending. Such a synaptic device is expected to be used as a vision photo-receptor.

## Introduction

The present day’s revolution is going to place for the automation of all dangerous tasks, such as working in extreme weather and toxic environments, war, fire, relief during natural disasters, space exploration missions, etc., using artificial intelligence (AI) based humanoid robots^1,2^. To use such robots effectively, a sensory and response system is very critical. A nociceptor in our neuronal system senses the noxious stimuli and responds accordingly with the instructions of the brain to minimize the potential physical damage^3^. Notably, during exposure to the noxious stimuli for a protracted duration, the nociceptor does not adapt to it, however, other sensory receptors (e.g., touch, hearing, smell, vision, and taste) abruptly or gradually reduce their sensitivity. For vision, senses by photoreceptors consist of rods and cones, where to managing the intensity of light is the responsibility of rods and cones are accountable for the colours, also known as photopic vision. Optical signals detected by the photoreceptors reached the brain through inhibitory synapses followed by the neuronal network. The development of electronic devices owning such sensory behaviour, which is similar to biological photoreceptors, will pave a vital way to obtaining electronic photo-receptors. This device can identify the external optical stimuli and transfer them to the internal nervous system^2^.

Memristor, the so-called fourth fundamental element, was predicted roughly a half-century back by Chua^4^. It becomes an emerging technology and participates in the rat race with others, such as FRAM (Ferroelectric Random Access Memory), MRAM (Magnetic Random Access Memory), and PCRAM (Phase Change Random Access Memory) a little more than a decade ago^5–11^. When MRAM and FRAM attract popularity with their performances for memory applications, a big question has arisen about the future of memristor technology. The memristor regains the limelight among the other emerging technologies as time passes. It was proving its applicability in several areas like neuromorphic computing, medical applications, robotics, space applications, 5G communications, etc., though it was initially limited to non-volatile memory applications^12–15^. To design the photo-receptor, the device is required to be transparent and for that wide band gap binary metal oxides, such as ITO (Indium Tin Oxide), AZO (Aluminium doped Zinc Oxide), are preferred to be transparent electrodes^16^.

Memristor-based synapses for applications in neuromorphic computing by triggering with electrical pulses make it more popular not only for high performances computing applications but also for use in biological purposes^16–18^. The conducting filaments are controlled using the electrical triggering for memristor synapse potentiation and depression properties. However, excitation increases in the memristor devices when such synaptic behaviours were controlled using the photonic pulses, which could be more beneficial in terms of processing speed, as the speed of light is ultimate and definite. In such cases, the device can consume low power along with the beneficiaries of the virtually unlimited bandwidth, elimination of electrical Joule heating, dodging of crosstalk interference, and the potential of functional integration relating to optical signal sensing, storing, and handling^19,20^. Under inspiring this, the photoelectric plasticity in the ZnO_1−x_/AlO_y_ heterostructure devices^21^, mimicking synaptic properties in IZO/IGZO structure using photons^22^ and photonic potentiation and electric habituation in MoS_2_ were demonstrated^23^. To develop artificial vision, an optoelectronic memristor and its synaptic behaviour are very important for its direct response to the optical stimulation alongwith it’s memory behaviour and real-time processing of sensory data and visual information^24^. Several binary oxides such as ZnO, HfO_2_, ZrO_2_, TiO_2_, etc. were used to make memristor-based synapses by achieving analogue current–voltage switching behaviour^17,18,25^. Although some of the oxides are used for the optoelectronic synapses, the studies of fully transparent memristor synapses are still very limited.

Considering process compatibility, all oxide-based hetero-structure devices are a bit ahead of any other hetero-structures attracting attention from the device engineers. However, all oxide-based flexible invisible photoreceptors and their application for photopic vision are not proposed yet. Overall, this study demonstrates a highly transparent, flexible, all-oxide-based heterostructure memristor-based synapse on the PEN substrate. The excellent synaptic properties of this device are attributed to its optoelectronic switching behaviour. Its flexibility is proven through sustaining at different bending radii. The application of the memristor synapse as photo-receptors is also demonstrated for the first time. The conduction mechanism of the devices is also proposed with the help of physical characteristics. This work is an innovative application of the all-oxide memristor synapse as a photo-receptor.

## Experimental

**Figure 1:**
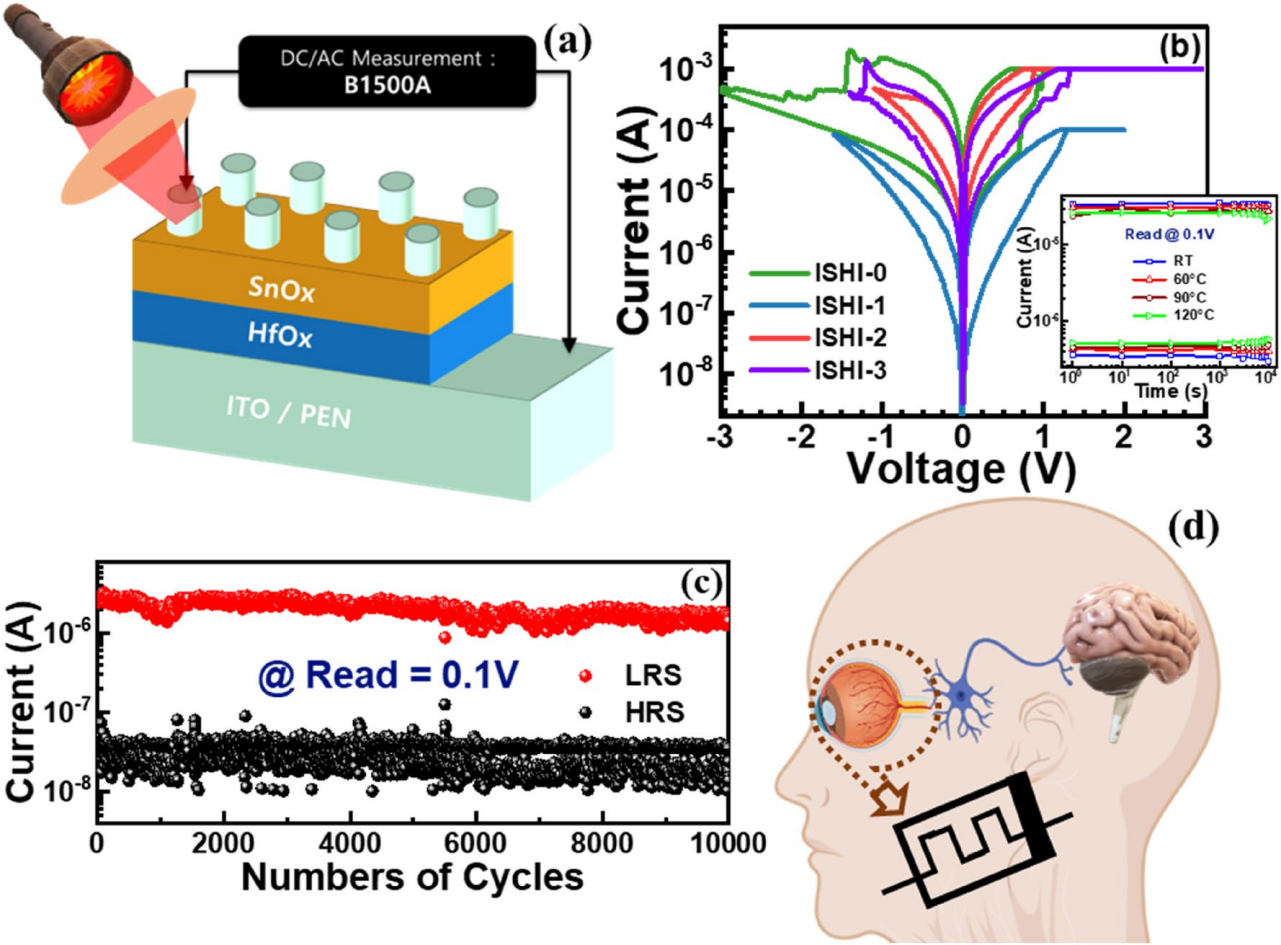
(**a**) Schematic architecture of our all-oxide optoelectronic synapse and photo-recepting measurement set-up using LASER sources. (**b**) Bipolar I–V set/reset characteristic of different memristor synapses, inset showing the retention of optimized ISHI-1 device at room temperature, 60 °C, 90 °C and 120 °C. (**c**) Endurance characteristics of the ISHI-1 and (**d**) schematic of the human photo-receptor.

Different thicknesses of HfO_x_ and SnO_x_ switching oxide thin films were sequentially reactive RF sputtered (Ar: O_2_ = 2:1) on commercial ITO as a bottom electrode (BE) coated PEN substrate. The thickness of the SnO_x_ film was optimized as 35 nm whereas those of HfO_x_ films were varied as 6, 10, and 15 nm, called ISHI-1, ISHI-2, and ISHI-3 (ISHI = ITO/SnOx/HfOx/ITO/PEN) devices, respectively. A control device without a SnO_x_ layer (ISHI-0) was also fabricated for comparison. The properties of those devices are presented in Table 1. Immediately after switching oxide deposition, a 180 nm thick ITO film was sputtered as a top electrode (TE) using a shadow mask (having 100 μm diameter). The schematic device structure is shown in Fig. 1a. Agilent B1500A semiconductor analyser equipped with a pulse signal generator was used to investigate the electrical switching properties of the devices. Blue (405 nm, SDL-405-LM-100T), Green (532 nm, SDL-532-LM-100T), and Red (633 nm, SDL-633-LM-100T) Light Emitting Diode (LED) sources were used to elucidate the photo-recepting behaviour. To study the optical transmittance spectra of the devices, a Hitachi U-3010 UV–visible spectrophotometer was used. For the structural and elemental analyses, cross-sectional high-resolution transmission electron microscopy (HRTEM) (JOEL, JEM-F200) equipped with energy dispersive X-ray (EDS) and X-ray photoelectron spectroscopy (XPS) (ULVAC-PHI PHI 5000 Versaprobe II) were employed.

**Table 1:** Comparison of linearity at LTP and LTD, conductance ratio, and the number of stable epochs of different ITO/SnO_x_/HfO_x_/ITO/PEN devices.

Thickness (nm) of SnO_x_/HfO_x_	Sample name	NL (LTP/LTD)	Conductance ratio	Epoch
35/0	ISHI-0	–	–	–
35/6	ISHI-1	1.53/1.46	1	350
35/10	ISHI-2	3.77/4.55	0.08	200
35/15	ISHI-3	2.79/2.71	0.08	50

## Results and discussion

Gradual switching is one of the primary signatures of a memristor for synaptic applications^13,26,27^. Figure 1b shows the various memristors' typical asymmetric bipolar I-V switching characteristics. Such asymmetries are quite expected from our asymmetric device structures. This phenomenon is also observed in the current–voltage bipolar switching curves of the devices having different SnO_x_ thicknesses of 10 nm, 20 nm and 35 nm using a 6 nm thin HfO_x_ layer, as shown in Supplementary Fig. S1. Among various SnO_x_ thicknesses, the 35 nm thick device shows better switching performance, as indicated in the supplementary Fig. S1c,f. However, the other devices suffered from abrupt switching and poor endurance, as shown in the supplementary Fig. S1a,b,d,e. The memristor having 35 nm SnO_x_ thickness and 6 nm HfO_x_ thickness (ISHI-1 device) exhibits not only improved gradual switching compared to other HfO_x_ and SnO_x_ thickness-based devices but also has low leakage current and can work at low compliance current (I_CC_) (100 μA) during forming (not shown here), making it suitable for low power application. However, a minimum of 1 mA I_CC_ is essential for the rest devices. The lower I_CC_ in the ISHI-1 device leads to its higher resistance in comparison to the other devices^28–31^. Although, the individual set/ reset voltages of ISHI-1 are slightly higher than some of the other devices. However, the optimised ISHI-1 memristor can retain perfectly stable LRS/HRS for more than 10^4^ s at room temperature as well as elevated temperature up to 90 °C, as shown in the inset of Fig. 1b. At 120 °C a slight degradation in R_off_/R_on_ is observed with time. It is indicated that an acceptable endurance of more than 10^4^ cycles with a 50X memory window increases the impact of the ISHI-1 device (Fig. 1c), whereas all other devices are suffering with poor switching stability, small memory windows, and limited endurance cycles. The comparison of different resistive switching parameters such as compliance current, set/ reset voltage, switching ratio, endurance and retention values at various thickness combinations of SnO_x_ and HfO_x_ layers are summarised in Table 2.

**Table 2:** Comparison of different resistive switching parameters at various thicknesses of SnOx and HfO_x_ layers of ITO/SnO_x_/HfO_x_/ITO/PEN devices.

SnO_x_:HfO_x_ (in nm)	I_CC_ (μA)	On/off ratio	Set/reset voltage (V)	Retention	Endurance
10:6	200	5	1.1/−1.2	10^2^	110
20:6	500	20	1.5/−1.3	10^3^	160
35:6	100	500	1.3/−1.6	10^4^	10^4^
35:10	1000	50	0.85/−1.1	~ 10^4^	800
35:15	1000	20	1.3/−1.4	~ 10^4^	600
35:0	1000	50	1/−3	~ 10^3^	400

The working principle of the biological photo-receptor and its analogy to its equivalent electronic counterpart is illustrated in Fig. 1d. In the human visual system, the photo-receptor is a special receptor of optical sensory neurons for the photopic vision that recognizes noxious optical stimuli. When an optical stimulus (such as different colours, Red, Green, and Blue) is detected through lenses by photoreceptors located at a free nerve ending inside the vitreous body, an electrical signal is produced and directed to the brain through the synapses^32^. After detecting the signal by the photoreceptor, the generated signal is transferred to inhibitory synapses followed by the neuronal network to communicate with the brain. The amplitude or other threshold parameters are compared in the brain and act accordingly. The feasibility of all the devices as an electronic synapse to mimic the useful synaptic functions along with storing data could be possible in our devices as we observed gradual current changes in Fig. 1b.

**Figure 2:**
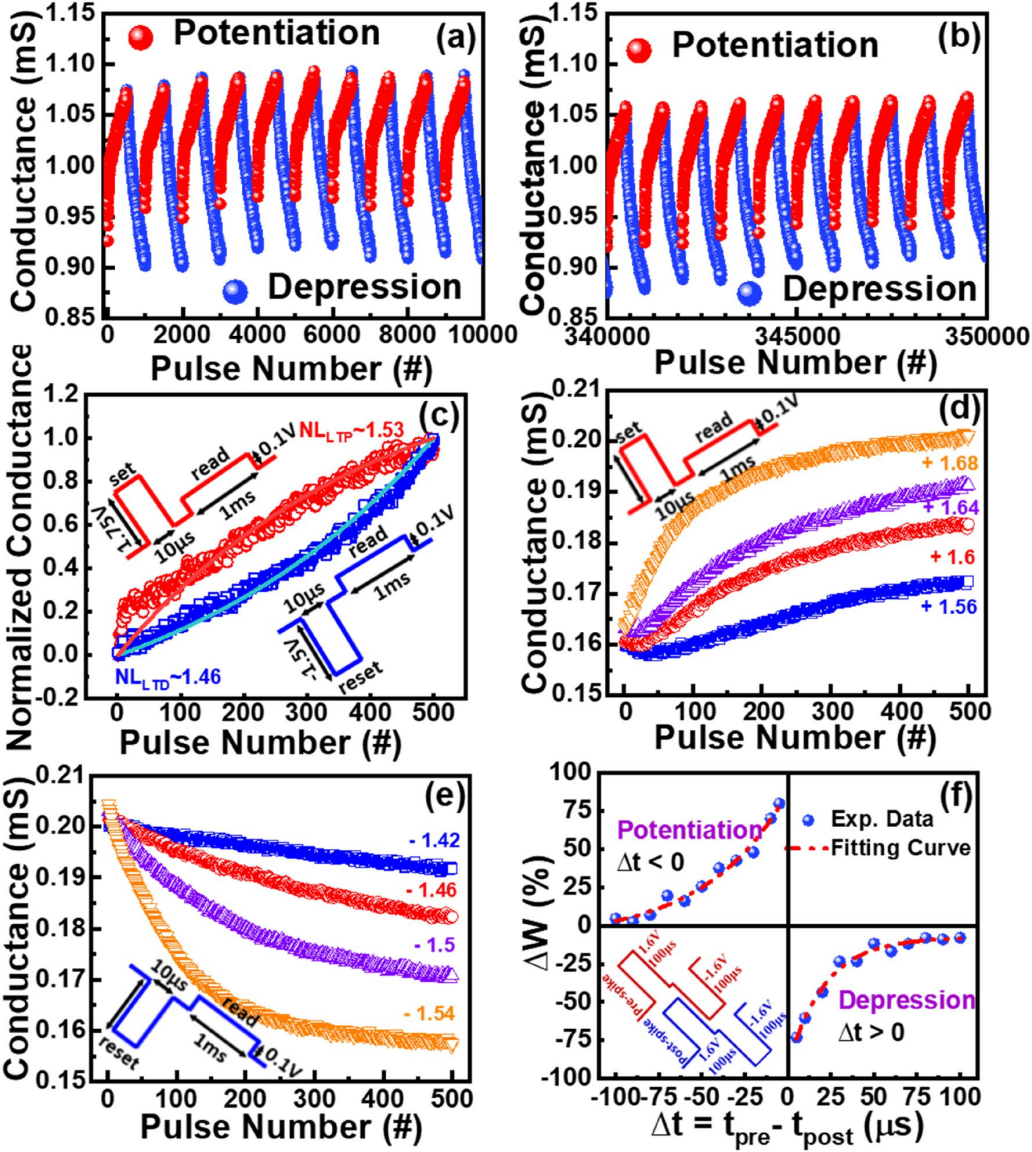
Pulse-induced LTP/LTD cycles of ISHI-1 device of (**a**) first and (**b**) last 20 cycles (10 epochs) with a total of 350 epochs. (**c**) Nonlinearity fitting of a typical LTP (red colour)/LTD (blue colour) cycle (inset showing the applied pulse scheme for potentiation and depression). Multi-level characteristics of the optimized ISHI-1 by controlling the pulse height for (**d**) potentiation and (**e**) depression. (**f**) Typical STDP learning behaviour of ISHI-1 synapse with the fitting curve, the pulse schemes for potentiation and depression presented in the third quadrant.

The pulse conductance is used to emulate the neuronal behaviour of the memristors. The neuronal behaviour of electronic synapses is represented as long-term potentiation/ depression (LTP/LTD) by changing the conductance states as a synaptic weight with the pulse as a spiking signal. Figure 2a,b indicate the first and last ten potentiation and depression epoch training, respectively, of the optimized ISHI-1 device. A stable 350 epochs training consisting of 500 pulses for each cycle is observed for this ISHI-1 device. Whereas, all other devices can only sustain up to 200 (ISHI-2) and 50 (ISHI-3) epochs training. To get the potentiation and depression characteristics, the optimized identical pulses of 1.6 and -1.46 V amplitude with 10 μs width for set and reset, respectively, and equal 0.1 V amplitude with 1 ms width read pulse width is applied to the ISHI-1 device, as shown in the inset of Fig. 2c. In addition to the excellent epoch training of ISHI-1 synapse, the device with the thinner (6 nm) HfOx film reduces the non-linearity (NL) values to 1.53 and 1.46 for potentiation and depression, respectively, as indicated in Fig. 2c. To find the nonlinearity values the potentiation and depression curves are simulated using Matlab codes based on the equations^17,18^

1 $${G}_{LTP}=B\left[1-{e}^{\left(\frac{P}{A}\right)}\right]+{G}_{min}$$

2 $${G}_{LTD}= -B\left[1-{e}^{\left(\frac{P-{P}_{max}}{A}\right)}\right]+{G}_{max}$$

3 $$\mathrm{Where},\mathrm{ B}=\frac{{G}_{max}-{G}_{min}}{1-{e}^{\frac{-{P}_{max}}{A}}}$$

where, G, P, and A are the conductance value, pulse number, and nonlinearity value, respectively. ${P}_{max}$ and ${G}_{max}$ are the experimental values of the maximum pulse and conductance, respectively. Table 1 presents the comparison of potentiation and depression NL values, conductance state ratio, and the total number of epochs of all the devices.

To increase the storage density of the device, the optimized memristor can also respond significantly to the multi-level conductance (MLC) states by gradually varying the synaptic weight with a step of 0.04 V from 1.56 to 1.68 V and −1.54 to −1.42 V with 10 μs pulse width, during potentiation and depression, as shown in Fig. 2d,e, respectively. In each conductance state, consecutive 500 AC pulses are applied with a 1 ms read time and 0.1 V read pulse. The ISHI-1 device appears linear conductance changes at smaller pulse heights while at higher pulse heights the logical conductance growth (shown in Fig. 2d,e) for both LTP and LTD**,** indicates a great capability of multi-level cell (MLC) applications**.** Moreover, the balance between dynamic range and conductivity linearity can be observed in the MLC study. The NL increases with the increasing pulse height as the conductance is rapidly saturated, which makes clear that the pulse height plays a crucial role in the practical synaptic operation.

A spiking Neural Network (SNN) is employed to confirm the application of memristor synapse. The Spike Timing-Dependent Plasticity (STDP) was investigated, which precisely maintained the balance between LTP and LTD in the Hebbian plasticity, and acted across the synapses depending on their neuronal activities between pre- and post-synapses (parent and child neurons respectively)^33^. The nature of the STDP curve is obtained from the conductance change (ΔG = G_LTP_ − G_LTD_) or synaptic weight (ΔW) during potentiation and depression and the pre-and post-synaptic signals (pulses) time difference (Δt). A pre-signal is applied at the TE and a post-signal at the BE, as schematically shown in the third quadrant of Fig. 2f. A spike pairing (amplitude is doubled than the pre-and post-spike after pairing) at different pulse widths (t_p_) attributes the STDP properties. To match the STDP result with the theoretical exponential nature, the synaptic weight needs to be precisely controlled, depending on the net pulses before (W_0_) and after (W_1_) pulse pairing, through the relation [ΔW = (W_1_ − W_0_)/W_0_]. The STDP adjusts the connection strength between the neurons based on the relative timing of a particular neuron's output and input action potentials (or spikes)^33^. The STDP property is proven for the learning algorithm in the neuronal network for pattern recognition and also in movement recognition as a Dynamic Vision Sensor (DVS)^34^.

As synaptic weight (ΔW) is pulse width (t_p_) independent^35,36^, optimized fixed 100 μs pulse width and ± 1.6 V double pulse amplitudes of rectangular wave signals (the pulse schematics are in the third quadrant of Fig. 2f) are applied to the ISHI-1 device for the STDP characterization and the result is depicted in Fig. 2f. The sequence of pre-and post-synaptic pulses defines the polarity of ΔW. For LTD, when post-spike follows pre-spike (Δt > 0), which decreases the synaptic weight (W_1_ > W_0_) by rupturing the conducting filament as the effective signal is negative. The reverse happens during LTP when the pre-spike follows the post-spike (Δt < 0). The STDP data for potentiation and depression is presented in the second and fourth quadrants of Fig. 2f, respectively, with the exponentially fitted (Eq. 4) curve^37^.

4 $$\Delta {\text{W}} = \left\{ {\begin{array}{*{20}l} {{\text{A}}_{ + } \exp ( - \Delta {\text{t}}/\uptau _{ + } ),\;\Delta {\text{t}} < 0} \hfill \\ {\quad \left( {{\text{A}}_{ + } \, = \, 96.22902\, \% \, \, \uptau _{ + } \, = \, - 47.80822\, {\text{ }}\upmu {\text{s}}} \right)} \hfill \\ {A_{ - } \exp ( - \Delta {\text{t}}/\uptau _{ - } ),\;\Delta {\text{t}} > 0} \hfill \\ {\quad \left( {{\text{A}}_{ - } \, = \, - 83.47906\% \, \, \uptau _{ - } \, = \, 21.51332\, {\text{ }}\upmu {\text{s}}} \right)} \hfill \\ \end{array} } \right.$$

where *A*_+_ and *A*_−_ refer to the maximum values of synaptic change and *τ*_+_ and *τ*_−_ are the range of pre- to post-synaptic spike time intervals over which synaptic strengthening and weakening are substantial. The values of A_+_ and A_−_, and *τ*_+_ and *τ*_−_ are obtained by fitting the experimental data with Eq. (1). When the Δt is + 5 and −5 μs, the Δw is maximum i.e., −73.12 and 79.91% for the potentiation and depression, respectively. The conductance change is inversely proportional to the time difference of the synapses similar to the biological synapse as reflected in Fig. 2f. The lower non-linearity coefficient from the STDP results of the ISHI-1 device makes it suitable for the neuromorphic application**.**

**Figure 3:**
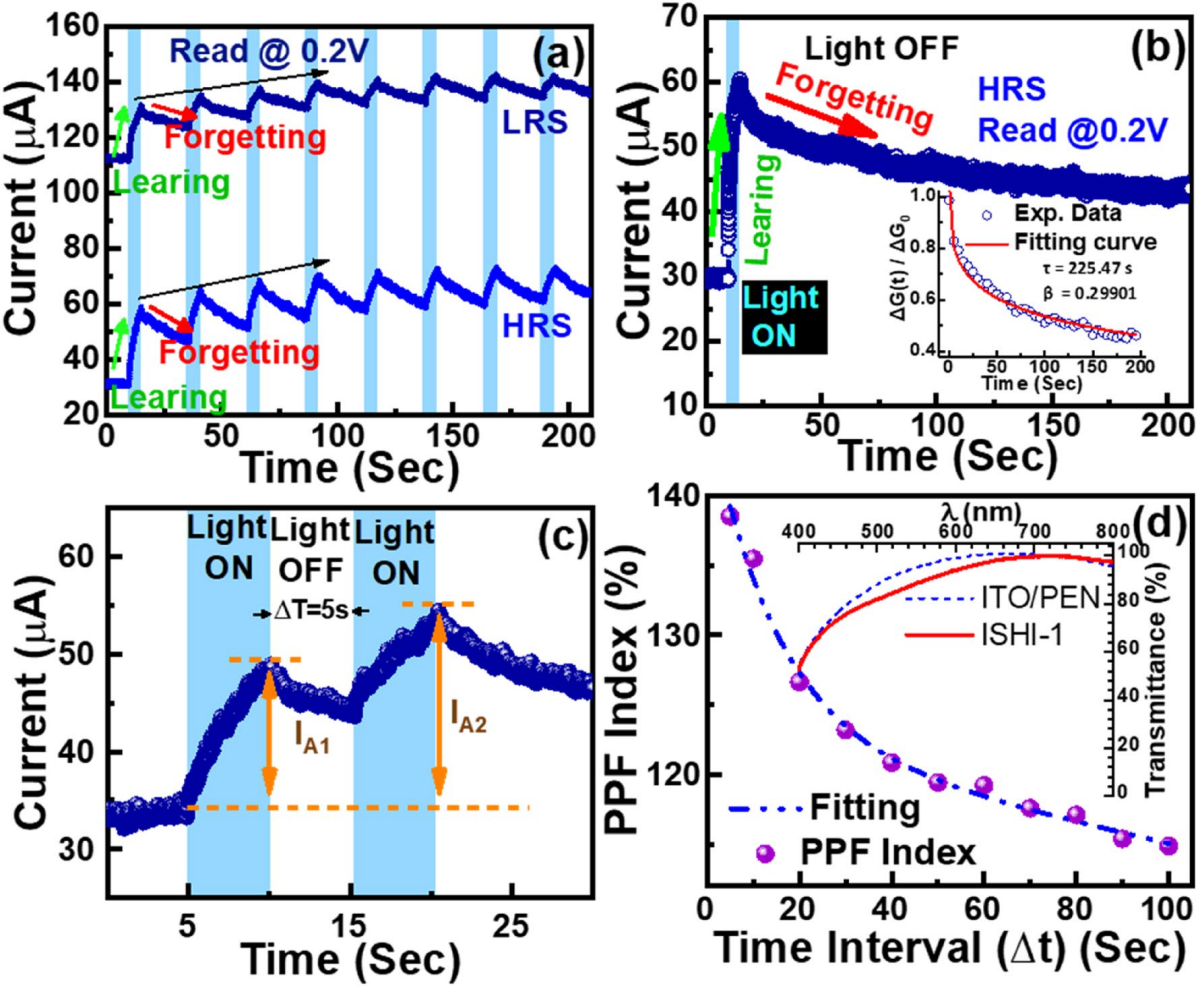
(**a**) Multi-level photocurrent states at LRS/HRS with typical blue light irradiation for 5 s followed by 20 s dark relaxation of eight cycles for ISHI-1 device. (**b**) Typical optoelectronic sensitivity at HRS after 5 s illumination and 200 s relaxation to achieve steady-state forgetting for the device. Inset shows the fitting result of the forgetting curve of the optoelectronic synapse during dark relaxation. (**c**) Pulse pair photo response under blue light irradiation, 5 s exposure with 5 s time intervals. (**d**) Photonic PPF index (I_A2_/I_A1_) with Δt between consecutive pulses with the simulated curve, inset showing the normalised transmittance spectra comparison of the ISHI-1 device with the commercial substrate.

To investigate the optoelectronic properties of the memristor synapse applied for biological photoreceptors, laser-induced optical signals instead of electrical signals are used. The optoelectronic sensitivity of the ISHI-1 device at LRS and HRS under blue light illumination at 0.2 V read voltage is shown in Fig. 3a. For the learning process, the device is illuminated for 5 s followed by 20 s dark relaxation, called forgetting. The responses of eight consecutive optical pulses for learning and forgetting are indicated in Fig. 3a. Continuous illumination for 5 s, raises the photo-current rapidly from 29.6 to 60.4 μA, increasing by ~ 104% and 160.9 μA to 195.4 μA, increasing by ~ 21% at HRS and LRS respectively. The remarkable effect at HRS indicates the wider implementation of the memory operation in this region.

To analyse the memory loss after applying the optical pulse, the device is illuminated for 5 s followed by 195 s for forgetting, as shown in Fig. 3b. The dark relaxation during forgetting can be explained using the modified Ebbinghaus forgetting model^38^ (Eq. 5) by Hu et al.^39^ (Eq. 6) of human psychology. The oxygen vacancy-based conductive filament model is considered by Hu et al. to explain the conduction mechanism of the memristor-based synapses, whereas the Ebbinghaus equation is similar to the discharge effect of the MIM capacitor, where completed charge–discharge takes place. All oxide-based memristors we investigated here are not completely discharged (70.9% retained after 5 s and 17.35% retained after 100 s time interval), as shown in Fig. 3a–c, due to the partial rupture of the conducting filament and the effect of photo-current. The Hu model considers the decay constant β (0 < β < 1) to analyse the memory loss from the forgetting curve as follows:

Ebbinghaus forgetting model ^38^:

5 $$R=\mathrm{exp}(-t//S)$$

Hu model ^39^:

6 $$P=\Delta \mathrm{G}(\mathrm{t})/\Delta {\mathrm{G}}_{0}=\mathrm{exp}{(- (t/\tau )}^{\beta })$$

where *R, S, t,* P, G(t), τ, and β are the memory content, memory stability*,* time, probability, conductance at time t, conductance relaxation time, and fitting index respectively. ΔG(t) = G(t) − G_i_, ΔG_0_ = G_0_ − G_i_, where, G_i_ and G_0_ are the initial conductance before and after stimulation, respectively. The learning and forgetting curves at HRS under blue light irradiation after an initial optical pulse are illustrated in Fig. 3b. The simulated result of photo-conductance decay of Fig. 3b using Eq. (6) during the dark relaxation of the ISHI-1 device is presented in the inset of Fig. 3b. The forgetting photo-conductance decay curve is fitted well at respective values of τ and β of 225.47 s and 0.29901. These values corroborate with the values of τ and β obtained from the human brain forgetting behaviour of 37 s and 0.31, respectively^40^. Remarkably, the value of β in our device is almost matched with that of the human brain. Such light sensitivity behaviour of the device enables its application to the biological system as an electronic synapse-based photo-receptor. It responded well to the optical stimuli, quite similar to the photo-receptor of human eyes.

For application in the biological system, paired-pulse facilitation (PPF) is a form of short-term synaptic plasticity which is obligatory to decode the optical or visual signal’s temporal properties. The ISHI-1 device is illuminated for 5 s with the blue light of two identical optical pulses by precisely tuning in-between time intervals (Δt = 5 s), as shown in Fig. 3c. The device is kept in the dark for a longer (10 s) duration for relaxation after the second pulse, as it’s not considered for the PPF index calculation. Figure 3c indicates that the photocurrent influenced by Δt after the second illumination, I_A2_, is greater than I_A1_ after the first illumination^41^. Figure 3a–c depict that the dark relaxation is significantly influenced by the time interval between two consecutive pulses. The temporal variation of the PPF index (I_A2_/I_A1_) (Fig. 3d) indicates the exponential decay of the PPF index from the highest 138% at 5 s to 115% at 110 s showing an inverse relationship with the time difference, corroborating with the biological synaptic behaviour. A two-phase exponential decay function is used to simulate the experimental data using the equation^42^,

7 $$PPF \left(\%\right)={\mathrm{C}}_{1}\mathrm{ exp}( -\mathrm{t}/ {\uptau }_{1}){+\mathrm{C}}_{2}\mathrm{ exp}( -\mathrm{t}/ {\uptau }_{2})$$

where C_1_ and C_2_ are the initial PPFs, the pulse pair time interval is t, and the relaxation time for slow and rapid decay phases are ${\uptau }_{1}$ and ${\uptau }_{2}$, respectively. The experimental results are best simulated at C_1_ = 22.99%, C_2_ = 23.434%, ${\uptau }_{1}$ = 14.627 s and ${\uptau }_{2}$=228.33 s. The relaxation time value corroborates with the value of the forgetting curve of optoelectronic synapse during dark relaxation as shown in inset of Fig. 3b. High transparency of more than 98% of the deposited device in the visible wavelength (380–750 nm) region is confirmed, as shown in the inset of Fig. 3d. The transmittance spectrum of the commercial ITO-coated PEN substrate is also presented for comparison. This optic-induced electrical behaviour accompanying by the high transmittance attribute that this device has the potential for use as a photo-receptors.

**Figure 4:**
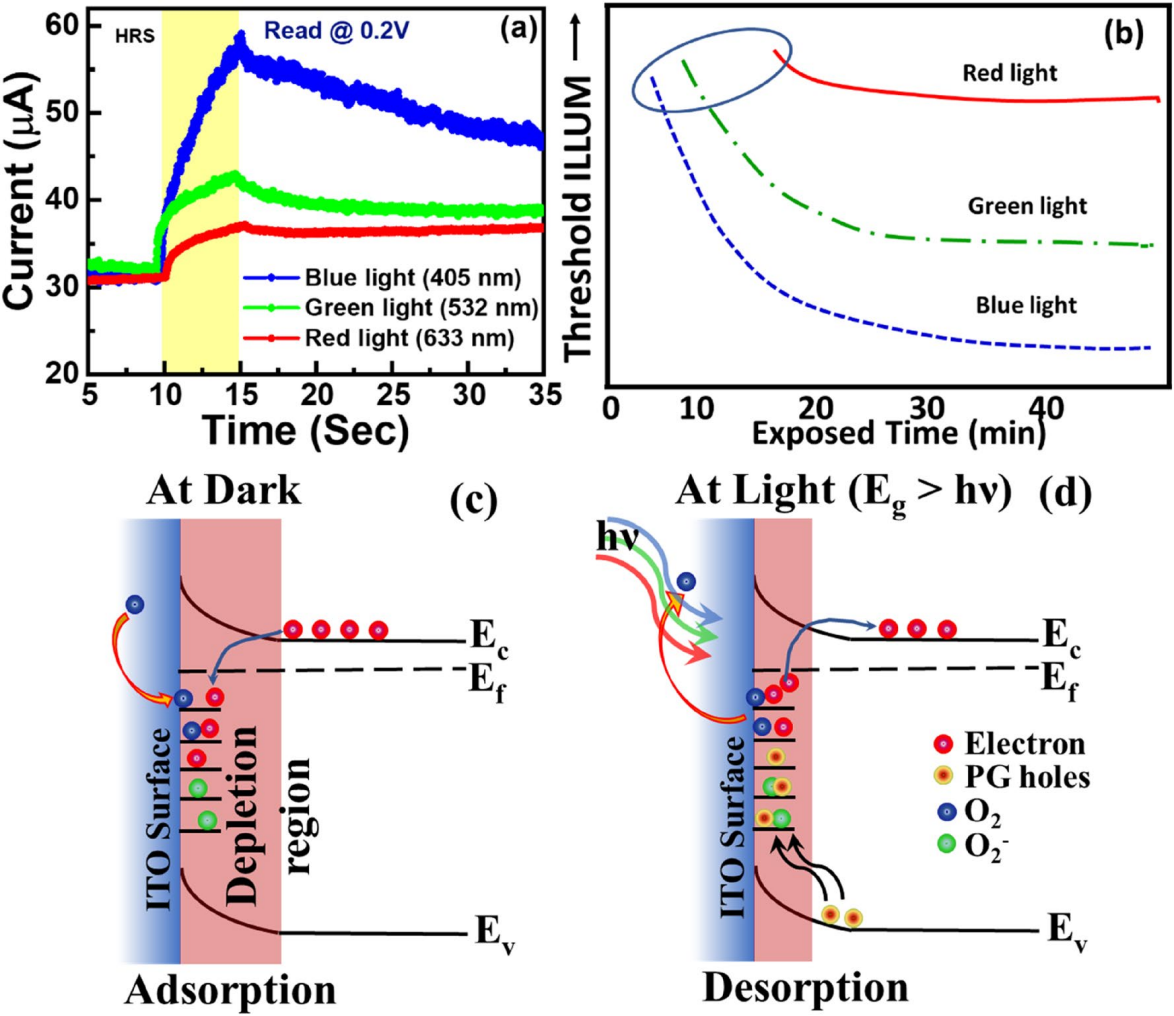
(**a**) Optoelectronic sensitivity of the ISHI-1 after being illuminated with three different wavelengths of light: blue, green and red at HRS after 5 s illumination. (**b**) Representative photopic vision sensitivity of the human eye^32^. Schematic band-diagram of carrier movement during (**c**) dark and (**d**) red, green and blue light irradiation. PG holes means photo-generated holes.

The sensitivity of the ISHI-1 under three different lights such as blue, green, and red in HRS at 0.2 V read voltage is presented in Fig. 4. It is indicated in Fig. 4a that the photocurrents after 5 s of blue, green, and red light irradiations are increased by 28.5 μA (93%), 11.6 μA (37%), and 6.3 μA (20%), respectively. The electron’s trapping and de-trapping by the oxygens on the trap states of ITO/ SnO_x_ surface is mainly responsible for the increase of current of the devices at lower energy light^43,44^. This photo-conduction is correlated with the adsorption and desorption of O_2_ at the ITO surface under dark and illumination (blue/red), respectively. In a dark environment, O_2_ are adsorbed at the surface and consumed electrons from the conduction band of n-type ITO to produce O^2−^ ions with the assistance of trapped free electrons, as shown in Fig. 4c. This adsorption attributes a larger depletion region at the ITO surface and induced upward band bending with reduced conductivity than the flat band condition^45^. After exposure to lower energy light than the bandgap of the material (~ 3.6 eV) like blue (λ = 405 nm; Energy = 3.06 eV), green (λ = 532 nm; Energy = 2.33 eV) or red (λ = 633 nm; Energy = 1.96 eV), the photogenerated holes are created as the hole density is comparatively less in the n-type semiconductor. These photogenerated holes are migrated towards the ITO surface and re-transformed into O_2_ from O^2−^ ions called the desorption process. Subsequently, the trapped electrons are photo-excited and returned to the conduction band (de-trapped) to participate in the conduction process, attributed a smaller depletion region with higher conductivity of ITO^44^. These de-trapped electrons act as a majority carriers for the conduction process, leads to photocurrent generation. Once the light is off the O_2_ is re-adsorbed on the ITO surface and the photo-current gradually decreases. Hence the electron’s trapping and de-trapping by the O_2_ on the ITO surface is mainly responsible for the change of current of the devices, when exposed by the lower energy light.

Enhanced photocurrent response from blue light in comparison to the rest colours for its higher light energy can excite more photo-generated electrons. Interestingly, as seen in Fig. 4a, the current sensitivity increases with the decrease of the wavelength of the light, quite similar trends to the light sensitivity for the wavelength with the photopic vision of the human eye^32^, as shown in Fig. 4b. It is shown that prolonged time takes to detect red colour light, whereas the least time for blue light, indicating that the sensitivity for red colour is lower than that of the blue colour. As the saturation current as well as response current of the device after illumination for 12 s, are higher for blue light than the red light, revealing the highest sensitivity with the blue light for photopic vision, which resembles the photopic vision of human vision. It confirms again that the ISHI-1 can be used as a photo-receptor of the vision system.

**Figure 5:**
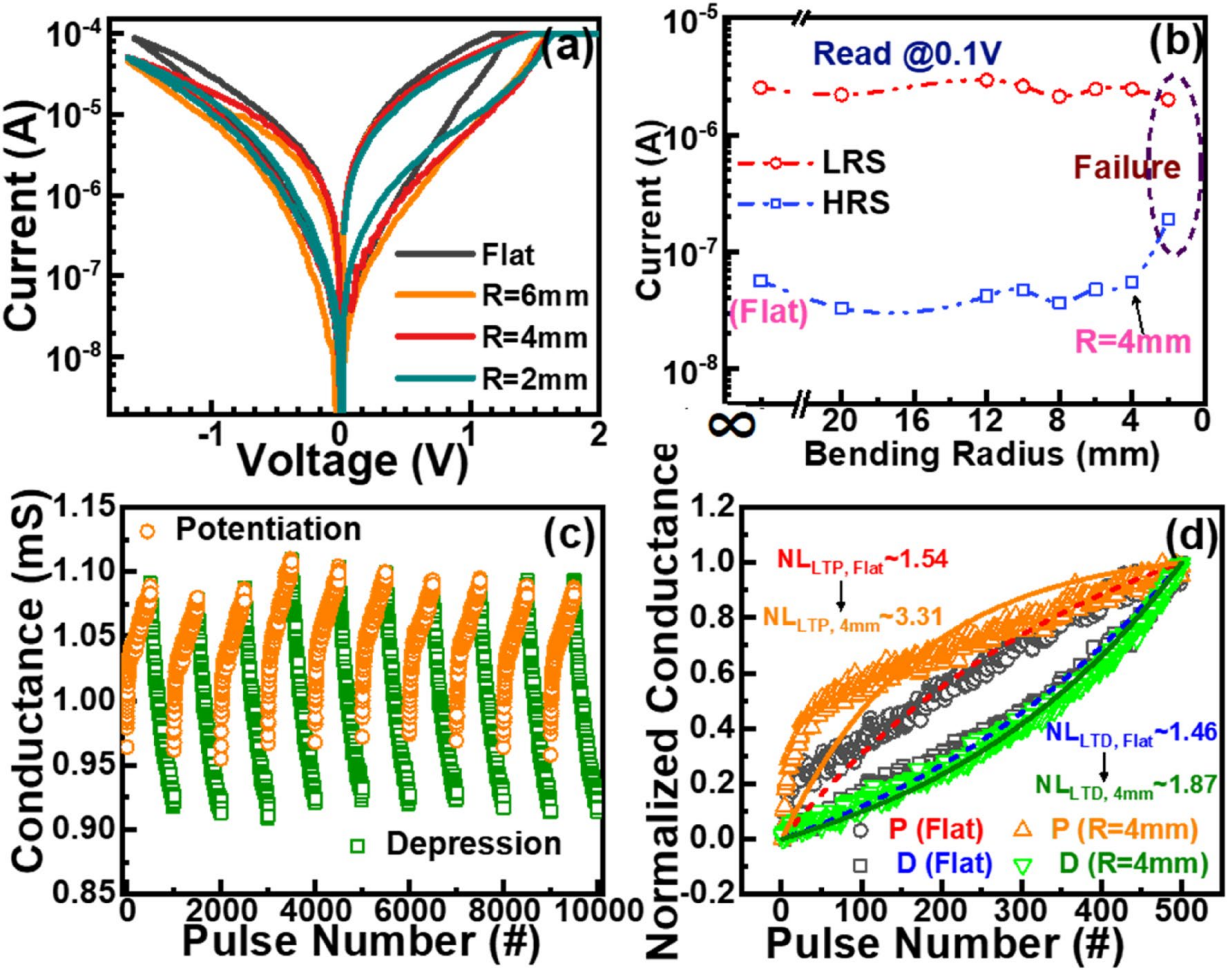
(**a**) Bipolar I–V curves at a different bending radius of the ISHI-1 device. (**b**) Variation of LRS and HRS with the bending radius. (**c**) 10 cycles of LTP/LTD after 4 mm bending and (**d**) nonlinear fitting comparison of a typical LTP/LTD of flat (dash line) with that after 4 mm bending.

To realize the device for the electronic flexible bio-synapse application, the flexibility of the device at different bending radii from a flat surface (bending radius ∞) to a 2 mm bending radius is studied and the result is given in Fig. 5a. It is noticed that the optimised device is suffered from the significant LRS/HRS change at a higher bending radius (R = 2 mm), whereas up to R = 4 mm bending a negligible bending effect indicates the ISHI-1 synaptic device can sustain well up to 4 mm bending radius. It is believed that the formation of a microcrack at a higher bending radius degrades the switching transport properties. Up to a 4 mm bending radius, this device can well maintain LRS/HRS ratio after continuous training pulses (Fig. 5b), with acceptable degradation of linearity compared with the same flat device, as shown in Fig. 5c. Although the linearity at LTP is affected more (N_LTP_: 1.53 → 3.31), in comparison with that at LTD (N_LTD_: 1.46 → 1.87), the device still maintains good linearity and consistency at its threshold bending radius, as shown in Fig. 5d. This phenomenon shows that the stress-induced bending creates strain at the switching interface affecting the filament formation process more than the ruptured filament. However, the ISHI-1 is still a potential candidate for the transparent wearable synaptic device.

**Figure 6:**
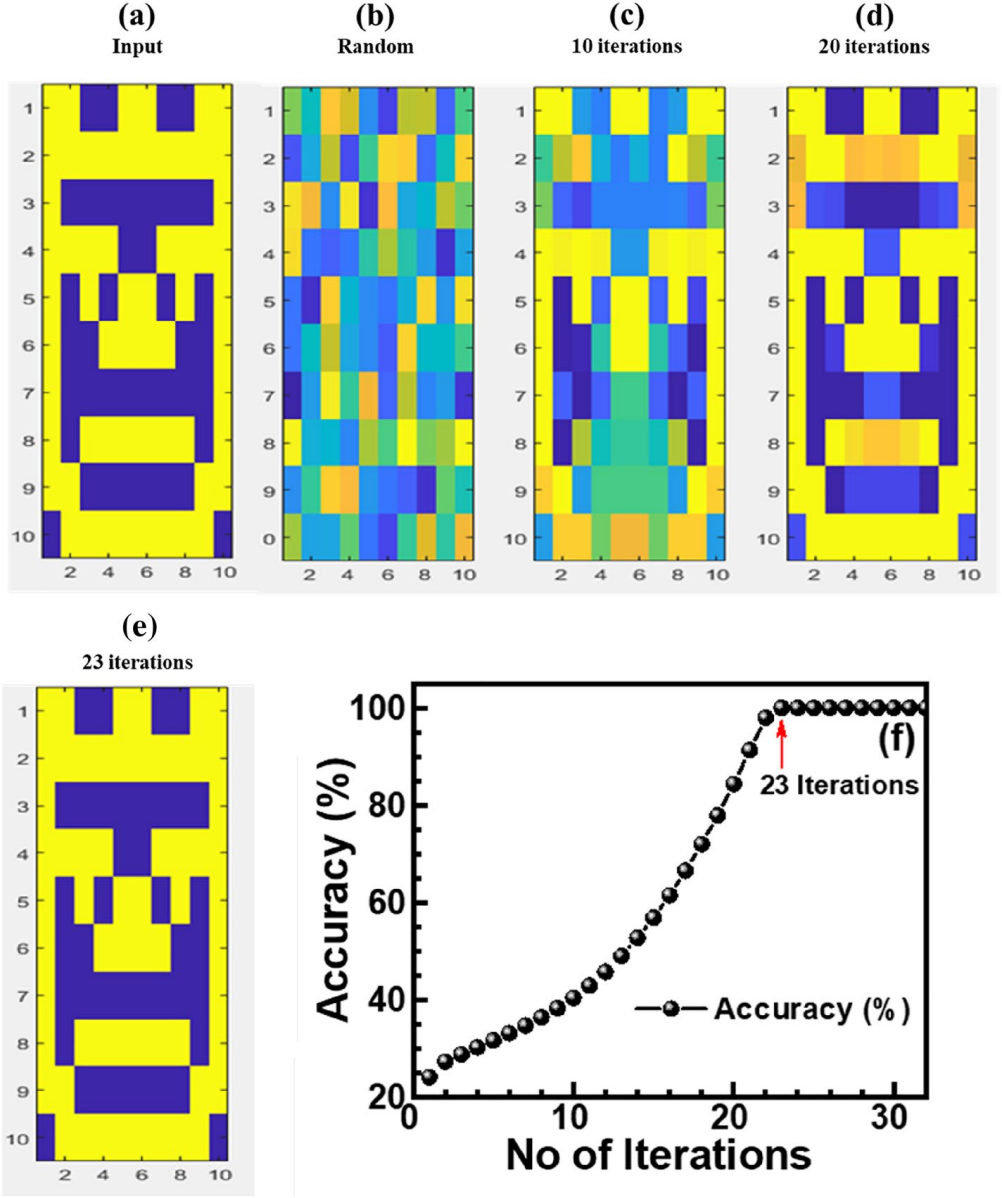
Training and recognition processes (**a**) input image, (**b**) random image, (**c**) image after 10 cycles, (**d**) image after 20 cycles, and (**e**) image after 23 cycles. (**f**) Change of recognition accuracy of ISHI-1 memristor with the number of iterations.

Pattern recognition is a crucial part of any synaptic device. The experimental LTP/LTD results (Fig. 2c) are simulated by adopting the Hopfield Neural Network (HNN) model^16,46^ for the pattern recognition scheme. It is depicted that the low LTP/LTD nonlinearity will give more recognition accuracy. The synaptic data are simulated using self-design MATLAB coding of a typical 10 × 10 pixels size input image (Fig. 6a). An HNN model with 100 synapses considers the normalized LTP/LTD conductance states in Fig. 2c as initial weights. The random and noisy image with very poor recognition (Fig. 6b) is observed at the initial stage due to the cluttering of neurons. However, by adding weights after 10 (Fig. 6c), 20 (Fig. 6d), and 23 training iterations (Fig. 6e), the improvement of pattern recognition becomes obvious with the learning process. This ISHI-1 device exhibits 84.34% recognition accuracy after 20 iterations and 100% accuracy barely after 23 iterations, showing high recognition speed for the synaptic application, as shown in Fig. 6f. The switching parameters and neuromorphic performances of different reported synapses are compared with those of our results in Table 3.

**Table 3:** Comparison of different resistive switching parameters of our results with the reported results.

Structure	Operating voltage (set/reset) (V)	Power (μW)	Retention	Endurance	P/D Cycles	P/D NL	Recognition accuracy	Light used	Photo conductance relaxation time (s) and fitting index	Ref
ITO/TaO_x_/ZTO/ITO	1.0/−1.2	100	10^4^	10^6^	790	1.8/−1.3	96%, 17 iterations	400 nm- 800 nm	–	^49^
Ni/ZTO/Si	3.0/−3.5	–	10^3^	–	3	–	89%	–	–	^50^
Ta/ZnSnO/TiN	−0.1/+ 1.5	100	10^4^	100	4	3.5/2.2	–	–	–	^51^
Ti/HfO_x_/HfO_2_/AlO_x_	0.9/−1	9	–	–	3	1.94/0.61	–	–	–	^52^
ITO/MoO_x_/Pd	2.5/−2.13	1	10^4^	–	500		98.6%,1000 epochs	365 nm		^24^
TiN/TaO_y_/TaO_x_/Pt	1 to 3 /−1.4 to −1.9	–	10^4^	1000	200	0.83/2.03	92%, 23 epochs	–	–	^53^
Au/BFMO/ITO/Glass	2.5/−2.5	50	3600	1000	1	0.29	98.80%	365 nm	–	^54^
Au/ZnO/Pt	2/−2	0.01	10^4^	–	20	–	98%, 40 epochs	530 nm	–	^55^
ITO/ZnO_x_/AlO_y_/Al	5 /−8	5	10^3^	1000	60	–	–	UV	–	^56^
ITO /MoO*x*/ Pd	2/-2	10^4^/cm^2^	800	–	–	–	98.6%, 1000 epochs	UV	–	^24^
Ni/TiW/HfO*x*/TaO*x*/TiN	1/−1	150	10^4^	200	35	3.54/1.65	100%, 24 iterations	–	–	^57^
Pt/TiO_*x*_/Al_2_O_3_/Pt/ITO	1.5/−0.4	10	10^4^	10^6^	–	1.62/1.46	91.15%, 100 epochs	–	–	^58^
Ta/TaOx/TiO2/Ti	–	–	–	–	50	1.85/−1.79	100%, 60 epochs	–	–	^59^
TiN/TaOy/TaOx/Pt	1.2/−1.5	0.7	10^4^	10^6^	200	0.83/-2.03	92%, 23 epochs	–	–	^60^
Ta/HfO2/Pt	0.5/−0.4	0.4	10^6^	10^3^	5	ANL 0.845	91%, 20 epochs	–	–	^61^
ITO/SnOx/HfOx/ITO/PEN	1.3/−1.6	0.27	> 10^4^	10^4^	350	1.53/1.46	100%, 23 iterations	Blue laser	225.47, 0.29901	This work

**Figure 7:**
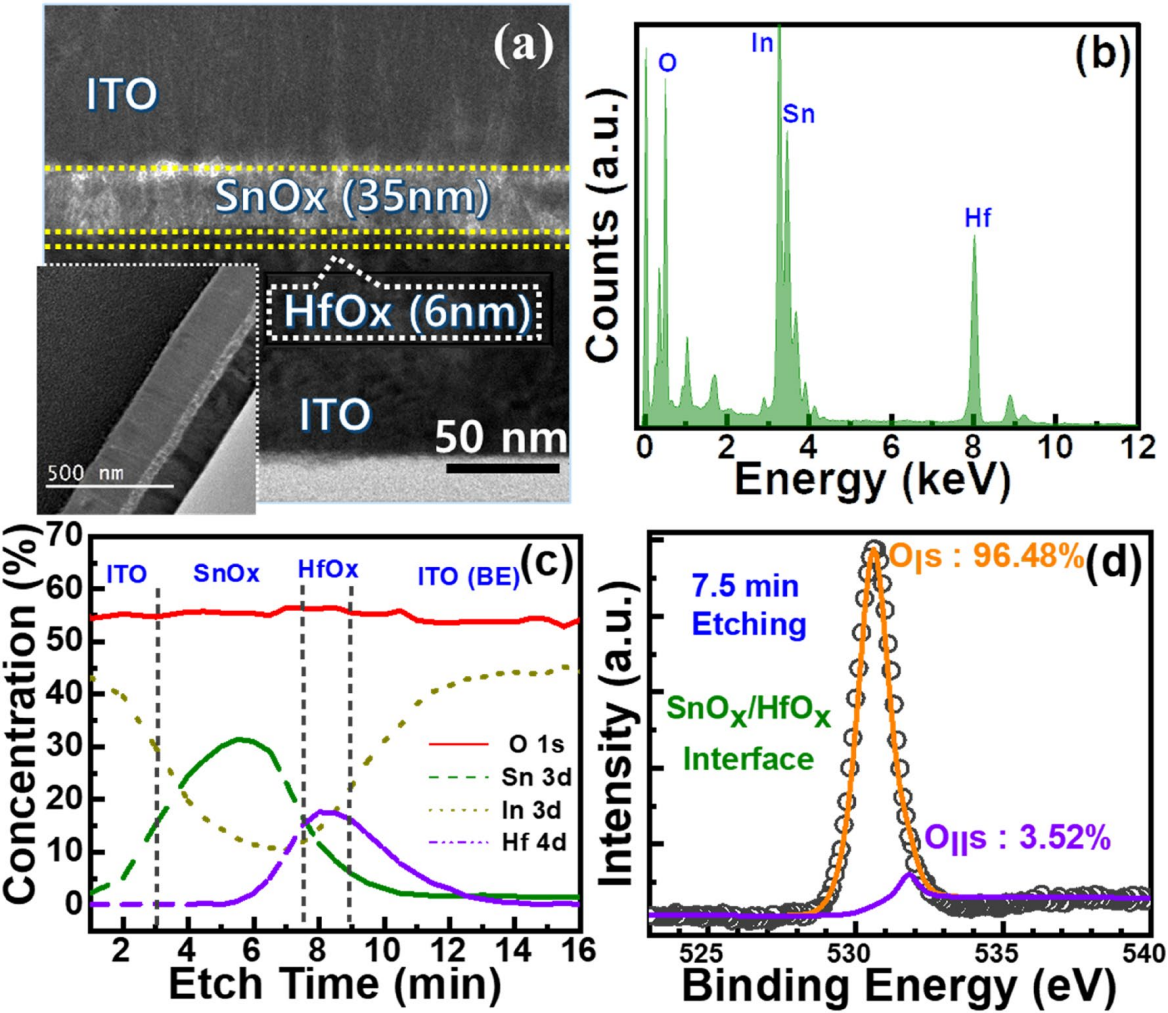
(**a**) TEM cross-section micrograph of the ISHI-1 memristor, inset showing the larger area of the device. (**b**) EDS elemental mapping spectra of the same device. XPS (**c**) depth profile spectra and (**d**) O1s fitting spectrum after 7.5 min etch time at the SnOx/HfOx interface of the device.

Figure 7a illustrates the cross-sectional TEM micrograph of the ISHI-1 memristor prior to the electrical measurements. A low-resolution image is shown in the inset of Fig. 7a. The formation of uniform interfacial layers is confirmed by the micrograph. Although the presence of Hf and Sn can be identified from the TEM EDS spectra (Fig. 7b), the XPS characterization is also carried out for the same sample to find the composition at different depths (Fig. 7c) to reveal the memristive mechanism. As composition plays a crucial role in the properties of the device and it attributes the switching mechanism based on the formation and rupture of conducting filaments by redox reactions. Not only clear signals of all materials at different depths are observed, but suppression of interfacial intermixing can also be realised from the XPS spectra of the ISHI-1 device, corroborating with the TEM results. To understand the oxygen vacancy concentration at HfO_x_ and SnO_x_ interface, the oxygen vacancies amounts are calculated from the oxygen spectra by resolving O_I_s and O_II_s peaks at 530.6 eV and 531.8 eV, respectively, where O_I_ and O_II_ correspond to lattice oxygen or oxygen ions and non-lattice oxygen or oxygen vacancies, respectively, as shown in Fig. 7d.

**Figure 8:**
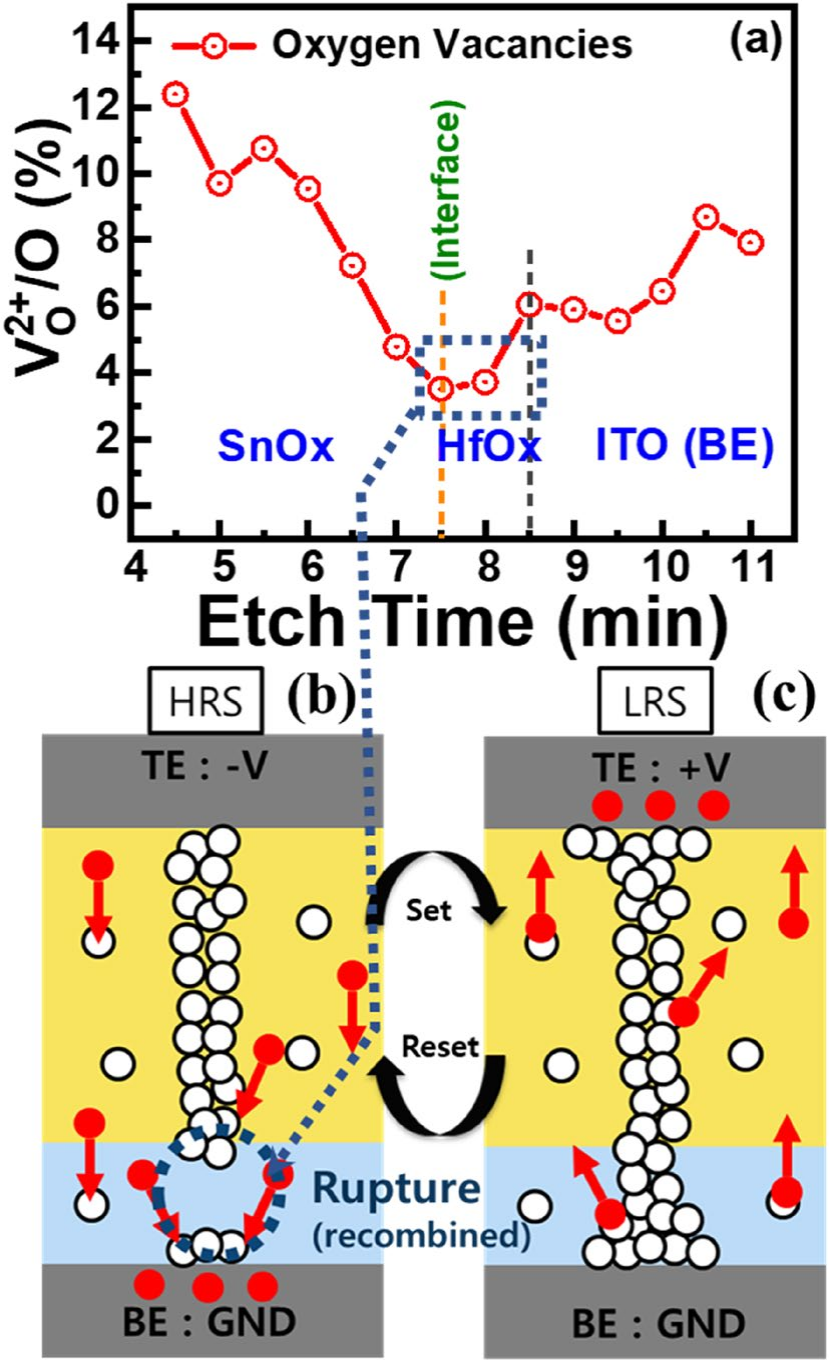
(**a**) Typical oxygen vacancies concentration ratio at different etching depths of ISHI-1 device obtained from XPS depth profile. Schematic resistive switching model during (**b**) reset and (**c**) set.

The variation of oxygen vacancies at different depths is summarized in Fig. 8a. Not only the higher amount of oxygen vacancies are present at the SnO_x_ layer, but also its decay tendency in Fig. 8a with the etching time indicate the possibilities of the presence of the filament residue at the SnO_x_ layer after the first reset. Hence, the filament formation and rupture process could be happened at the SnO_x_/HfO_x_ interface, as illustrated schematically in Fig. 8b,c. The presence of filament residue at the SnO_x_ layer is due to its thermodynamic stability and controlling the filament growth laterally and vertically, giving improved synaptic properties. This can be explained by the Gibbs free energy (GFE), as the GFE of HfO_2_ (~ −1145 kJ/mol) is lower than that of SnO_2_ (~ −520 kJ/mol)^47^, hence, HfO_2_ is more stable than SnO_2_ leading to the higher amount of oxygen vacancies are formed in SnO_x_ after forming and set^48^. During the set or writing process by applying a suitable positive bias at TE, the negatively charged field-induced oxygen ions drift toward the TEs and are stored there, while the positively charged oxygen vacancies gradually moved toward the BE, leading to the formation of oxygen vacancies based conductive filaments and the device switched from HRS to LRS, as shown in Fig. 8c. During reset, the filament would rupture at the interface and leave residual filament at the SnO_x_ layer due to its higher concentration of oxygen vacancies. The gradual I–V resistive switching shown in Fig. 1b might further prove the retaining of conductive filament at the SnO_x_ layer during reset, corroborating with the XPS results.

## Conclusions

The improved switching and synaptic properties of the device are based on the behaviour of the SnO_x_/HfO_x_ double layer, confirmed by the electrical measurements. The gradual switching with sustainable endurance and retention of 10^4^ cycles and 10^4^ s, respectively, were observed in the optimised device. The sufficiently long and stable training of 350 epochs with remarkable non-linearity of 1.53 and 1.46 for LTP and LTD, respectively, along with STDP properties makes the device suitable for the neuronal application. The optical sensitivity of red light is lower compared with that of blue light finding similarities with the photopic vision of the human eye. A light-induced synaptic switching with biological PPF index leads to the device being suitable for photo-recepting synapse application. High transparency of ~ 97% in the visible region with high flexibility up to 4 mm bending cause the device suitable for transparent and flexible electronics. After 23 iterations, the memristor synapse exhibits 100% accuracy from the HNN simulation indicating fast and accurate image recognition. The oxygen vacancies-based switching mechanism of the device is supported by the result of XPS analyses. This work gives light to the novel application of the memristor as an electronic photo-receptor for the neuromorphic visual system.

## Supplementary Information

Supplementary Figure S1.

## Data Availability

The datasets generated and/or analysed during the current study are not publicly available due to confidentiality but are available from the corresponding author upon reasonable request.
