# Supplementary material for: All oxide based flexible multi-folded invisible synapse as vision photo-receptor
Source: Sci Rep. 2023 Jan 26;13:1454. doi: 10.1038/s41598-023-28505-3 (PMC9880003; doi:10.1038/s41598-023-28505-3)
Supplement: Supplementary file 1 — Supplementary Figure S1. [file 41598_2023_28505_MOESM1_ESM.pdf]

All Oxide Based Flexible Multi-Folded Invisible Synapse as Vision Photo-receptor

Ping-Xing Chen,[a] Debashis Panda, [a,b]* and Tseung-Yuen Tseng [a]

[a] Institute of Electronics, National Yang Ming Chiao Tung University, Hsinchu City 30010, Taiwan

[b] Department of Electronics and Communication Engineering, CV Raman Global University, Bhubaneswar 752054, India.

*Corresponding author: phy.dpanda@gmail.com; ece.dpanda@nycu.edu.tw; ece.dpanda@cgu-odisha.ac.in

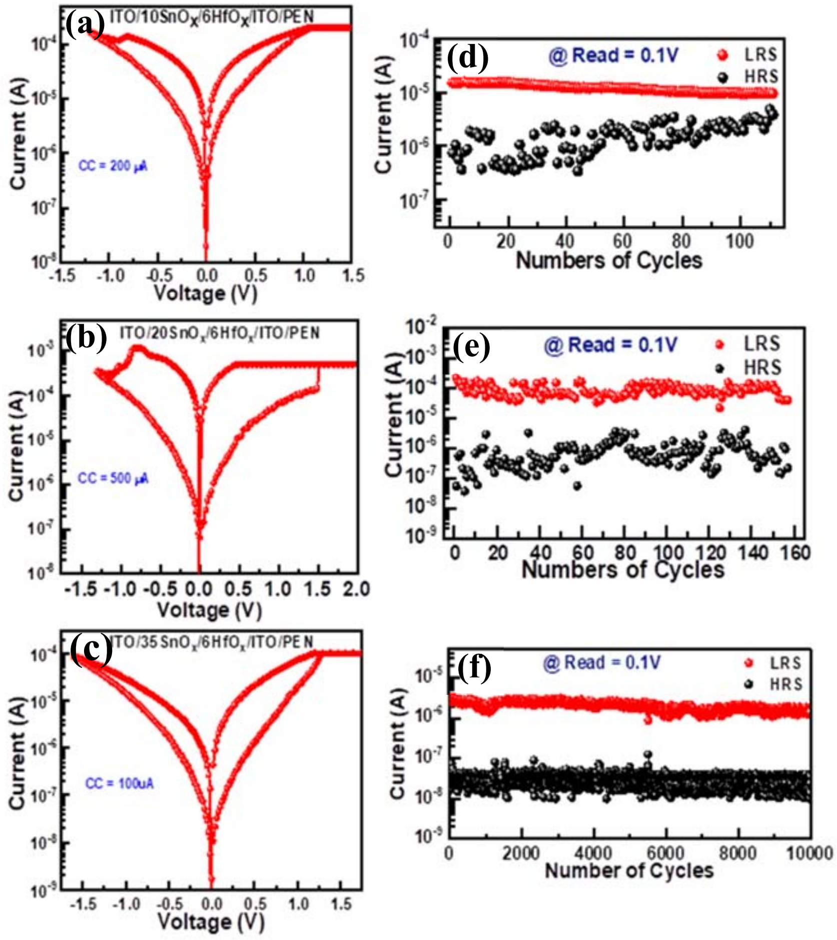

Figure S1: (a), (b) and (c): The optimization of the current-voltage bipolar switching curves of different SnO_x thicknesses of 5, 20 and 35 nm devices having 6 nm HfO_x layer for each device ; (e), (f) and (g) are the corresponding endurance results, respectively. The minimum compliance current is used to obtain the best switching and endurance performances. The 35 nm thick SnO_x layer device can sustain well for more than 10000 cycles along with gradual switching at a low compliance current.
